# 1,2-Bis(2-meth­oxy-6-formyl­phen­oxy)ethane

**DOI:** 10.1107/S1600536810054085

**Published:** 2011-01-08

**Authors:** Hongqi Li, Li Cai, Dongling Chen, Jinxing Li, Yijun Chen

**Affiliations:** a Key Laboratory of Science & Technology of Eco-Textiles, Ministry of Education, College of Chemistry, Chemical Engineering & Biotechnology, Donghua University, Shanghai 201620, People’s Republic of China

## Abstract

In the title compound [systematic name: 3,3′-dimethoxy-2,2′-(ethane-1,2-diyldioxy)dibenzaldehyde], C_18_H_18_O_6_, prepared from 1,2-dibromo­ethane and *ortho*-vanillin in the presence of sodium carbonate, the two vanillin units are linked *via* a CH_2_–CH_2_ bridge. The two benzene rings are inclined at a dihedral angle of 41.6 (5)°.

## Related literature

For the use of open chain-ionophores, including polyethyl­ene glycols, as microbiological agents and in ion binding, see: Valeur *et al.* (1992 ▶); Tuncer & Erk (2000 ▶). For the synthesis, see: Tuncer & Erk (2000 ▶). For related structures, see: Higham *et al.* (2010 ▶).
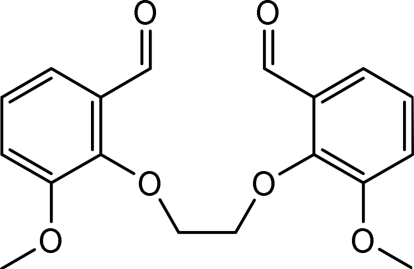


## Experimental

### 

#### Crystal data

C_18_H_18_O_6_
*M* *_r_* = 330.32
Monoclinic, 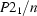

*a* = 4.161 (3) Å
*b* = 30.155 (18) Å
*c* = 12.934 (8) Å
β = 96.817 (7)°
*V* = 1611.6 (17) Å^3^
*Z* = 4
Mo *K*α radiation
μ = 0.10 mm^−1^
*T* = 296 K
0.12 × 0.10 × 0.08 mm

#### Data collection

Bruker APEXII CCD diffractometer
Absorption correction: multi-scan (*SADABS*; Sheldrick, 2004 ▶) *T*_min_ = 0.988, *T*_max_ = 0.992
14774 measured reflections
2815 independent reflections
1519 reflections with *I* > 2σ(*I*)
*R*_int_ = 0.096

#### Refinement

*R*[*F* ^2^ > 2σ(*F* ^2^)] = 0.060
*wR*(*F* ^2^) = 0.141
*S* = 1.00
2815 reflections
220 parameters
H-atom parameters not refined
Δρ_max_ = 0.18 e Å^−3^
Δρ_min_ = −0.18 e Å^−3^

### 

Data collection: *APEX2* (Bruker, 2004 ▶); cell refinement: *SAINT-Plus* (Bruker, 2004 ▶); data reduction: *SAINT-Plus*; program(s) used to solve structure: *SHELXS97* (Sheldrick, 2008 ▶); program(s) used to refine structure: *SHELXL97* (Sheldrick, 2008 ▶); molecular graphics: *SHELXTL* (Sheldrick, 2008 ▶); software used to prepare material for publication: *SHELXTL*.

## Supplementary Material

Crystal structure: contains datablocks global, I. DOI: 10.1107/S1600536810054085/sj5080sup1.cif

Structure factors: contains datablocks I. DOI: 10.1107/S1600536810054085/sj5080Isup2.hkl

Additional supplementary materials:  crystallographic information; 3D view; checkCIF report

## Figures and Tables

**Fig. 1.**
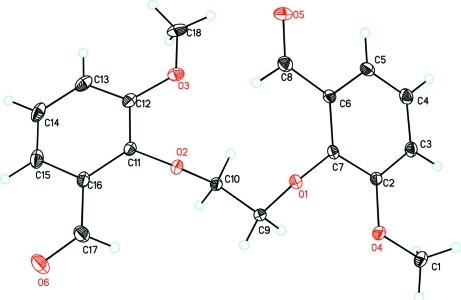
A view of the molecule of the title compound. Displacement ellipsoids are drawn at the 30% probability level and H atoms are shown as small spheres of arbitrary radii.

## supplementary crystallographic information

### Comment

Open chain ionophores including polyethylene glycols have proved to be extremely interesting compounds due to their versatility as microbiological agents and in ion binding (Valeur *et al.*, 1992). Their extraordinary capacity for ion binding has attracted much attention in view of their acyclic and bulky structures. For example, aromatic carbonyl derivatives of glycols such as 1,2-bis(2-methoxy-6- formylphenoxy)ethane were investigated to determine the role of sodium ions using steady state fluorescence spectroscopy (Tuncer & Erk, 2000). 1,2-Bis(2-methoxy-6-formylphenoxy)ethane and its analogues have also been used in the synthesis of dienone-ether macrocycles displaying molecular and supramolecular diversity (Higham *et al.*, 2010). Herein we present the single-crystal structure of the title compound.

### Experimental

The title compound was prepared as reported in the literature (Tuncer & Erk, 2000). Single crystals suitable for X-ray diffraction measurement was obtained by slow evaporation of the solution in acetone [m.p. 391–393 K; literature value: 392 K (Tuncer & Erk, 2000)].

### Refinement

All H atoms were placed at calculated positions and refined using a riding model approximation, with C—H = 0.93–0.97 Å and with *U*_iso_(H) = 1.2*U*_eq_(C) for aromatic and CH_2_ groups and = 1.5*U*_eq_(C) for methyl groups.

### Crystal data

**Table:**

C_18_H_18_O_6_	*F*(000) = 696
*M**_r_* = 330.32	*D*_x_ = 1.361 Mg m^−^^3^
Monoclinic, *P*2_1_/*n*	Melting point = 391–393 K
Hall symbol: -P 2yn	Mo *K*α radiation, λ = 0.71073 Å
*a* = 4.161 (3) Å	Cell parameters from 1432 reflections
*b* = 30.155 (18) Å	θ = 2.6–19.0°
*c* = 12.934 (8) Å	µ = 0.10 mm^−^^1^
β = 96.817 (7)°	*T* = 296 K
*V* = 1611.6 (17) Å^3^	Block, colorless
*Z* = 4	0.12 × 0.10 × 0.08 mm

### Data collection

**Table:**

Bruker APEXII CCD diffractometer	2815 independent reflections
Radiation source: fine-focus sealed tube	1519 reflections with *I* > 2σ(*I*)
graphite	*R*_int_ = 0.096
φ and ω scans	θ_max_ = 25.0°, θ_min_ = 2.6°
Absorption correction: multi-scan (*SADABS*; Sheldrick, 2004)	*h* = −4→4
*T*_min_ = 0.988, *T*_max_ = 0.992	*k* = −35→35
14774 measured reflections	*l* = −15→15

### Refinement

**Table:**

Refinement on *F*^2^	Secondary atom site location: difference Fourier map
Least-squares matrix: full	Hydrogen site location: inferred from neighbouring sites
*R*[*F*^2^ > 2σ(*F*^2^)] = 0.060	H-atom parameters not refined
*wR*(*F*^2^) = 0.141	*w* = 1/[σ^2^(*F*_o_^2^) + (0.059*P*)^2^] where *P* = (*F*_o_^2^ + 2*F*_c_^2^)/3
*S* = 1.00	(Δ/σ)_max_ = 0.001
2815 reflections	Δρ_max_ = 0.18 e Å^−^^3^
220 parameters	Δρ_min_ = −0.18 e Å^−^^3^
0 restraints	Extinction correction: *SHELXL*, Fc^*^=kFc[1+0.001xFc^2^λ^3^/sin(2θ)]^-1/4^
Primary atom site location: structure-invariant direct methods	Extinction coefficient: 0.0130 (19)

### Special details

**Table:**

Geometry. All e.s.d.'s (except the e.s.d. in the dihedral angle between two l.s. planes) are estimated using the full covariance matrix. The cell e.s.d.'s are taken into account individually in the estimation of e.s.d.'s in distances, angles and torsion angles; correlations between e.s.d.'s in cell parameters are only used when they are defined by crystal symmetry. An approximate (isotropic) treatment of cell e.s.d.'s is used for estimating e.s.d.'s involving l.s. planes.
Refinement. Refinement of *F*^2^ against ALL reflections. The weighted *R*-factor *wR* and goodness of fit *S* are based on *F*^2^, conventional *R*-factors *R* are based on *F*, with *F* set to zero for negative *F*^2^. The threshold expression of *F*^2^ > σ(*F*^2^) is used only for calculating *R*-factors(gt) *etc*. and is not relevant to the choice of reflections for refinement. *R*-factors based on *F*^2^ are statistically about twice as large as those based on *F*, and *R*- factors based on ALL data will be even larger.

### Fractional atomic coordinates and isotropic or equivalent isotropic displacement parameters (Å^2^)

**Table:**

	*x*	*y*	*z*	*U*_iso_*/*U*_eq_	
C1	0.3389 (9)	0.03026 (11)	0.5936 (2)	0.0647 (10)	
H1A	0.5683	0.0344	0.5956	0.097*	
H1B	0.2895	−0.0008	0.5895	0.097*	
H1C	0.2676	0.0424	0.6556	0.097*	
C2	0.2236 (7)	0.09709 (10)	0.4969 (2)	0.0448 (8)	
C3	0.4057 (8)	0.12260 (11)	0.5701 (2)	0.0554 (9)	
H3	0.5160	0.1092	0.6286	0.066*	
C4	0.4264 (8)	0.16785 (12)	0.5577 (3)	0.0622 (10)	
H4	0.5543	0.1845	0.6071	0.075*	
C5	0.2608 (8)	0.18851 (11)	0.4733 (3)	0.0547 (9)	
H5	0.2717	0.2192	0.4666	0.066*	
C6	0.0754 (7)	0.16342 (10)	0.3975 (2)	0.0453 (8)	
C7	0.0586 (7)	0.11786 (10)	0.4081 (2)	0.0420 (8)	
C8	−0.1070 (8)	0.18701 (12)	0.3090 (3)	0.0612 (10)	
H8	−0.2209	0.1703	0.2564	0.073*	
C9	0.0325 (8)	0.06410 (9)	0.2724 (2)	0.0461 (8)	
H9A	−0.1130	0.0407	0.2449	0.055*	
H9B	0.2118	0.0504	0.3156	0.055*	
C10	0.1595 (7)	0.08728 (10)	0.1841 (2)	0.0438 (8)	
H10A	0.2860	0.1129	0.2094	0.053*	
H10B	0.2979	0.0675	0.1501	0.053*	
C11	−0.0342 (7)	0.11450 (10)	0.0157 (2)	0.0440 (8)	
C12	0.1182 (8)	0.15543 (11)	0.0050 (3)	0.0532 (9)	
C13	0.1759 (9)	0.16920 (13)	−0.0933 (3)	0.0714 (11)	
H13	0.2742	0.1965	−0.1016	0.086*	
C14	0.0882 (11)	0.14262 (16)	−0.1793 (3)	0.0814 (13)	
H14	0.1269	0.1523	−0.2450	0.098*	
C15	−0.0523 (10)	0.10304 (14)	−0.1689 (3)	0.0747 (12)	
H15	−0.1064	0.0854	−0.2274	0.090*	
C16	−0.1190 (8)	0.08786 (11)	−0.0705 (2)	0.0526 (9)	
C17	−0.2777 (9)	0.04495 (12)	−0.0610 (3)	0.0703 (11)	
H17	−0.3183	0.0360	0.0050	0.084*	
C18	0.3642 (9)	0.21970 (10)	0.0892 (3)	0.0809 (12)	
H18A	0.5724	0.2134	0.0677	0.121*	
H18B	0.3920	0.2335	0.1565	0.121*	
H18C	0.2469	0.2393	0.0398	0.121*	
O1	−0.1379 (5)	0.09317 (6)	0.33650 (14)	0.0448 (6)	
O2	−0.1126 (5)	0.10109 (6)	0.11105 (14)	0.0446 (6)	
O3	0.1872 (6)	0.17924 (7)	0.09468 (19)	0.0640 (7)	
O4	0.1771 (5)	0.05236 (7)	0.50439 (15)	0.0560 (6)	
O5	−0.1154 (7)	0.22701 (8)	0.30153 (19)	0.0849 (9)	
O6	−0.3593 (8)	0.02042 (9)	−0.1333 (2)	0.1053 (11)	

### Atomic displacement parameters (Å^2^)

**Table:**

	*U*^11^	*U*^22^	*U*^33^	*U*^12^	*U*^13^	*U*^23^
C1	0.080 (3)	0.067 (2)	0.045 (2)	0.001 (2)	−0.0003 (19)	0.0127 (18)
C2	0.052 (2)	0.045 (2)	0.0370 (18)	−0.0069 (17)	0.0063 (15)	−0.0016 (16)
C3	0.059 (2)	0.066 (2)	0.039 (2)	−0.0036 (19)	−0.0018 (17)	−0.0019 (18)
C4	0.069 (3)	0.067 (3)	0.050 (2)	−0.018 (2)	0.0009 (19)	−0.0127 (19)
C5	0.060 (2)	0.047 (2)	0.058 (2)	−0.0068 (17)	0.0130 (19)	−0.0113 (18)
C6	0.050 (2)	0.044 (2)	0.043 (2)	0.0017 (16)	0.0124 (16)	−0.0041 (16)
C7	0.0433 (19)	0.050 (2)	0.0340 (18)	−0.0050 (16)	0.0084 (15)	−0.0083 (16)
C8	0.068 (2)	0.061 (3)	0.056 (2)	0.013 (2)	0.0077 (19)	−0.0067 (19)
C9	0.061 (2)	0.0381 (18)	0.0395 (18)	0.0023 (16)	0.0051 (16)	0.0010 (14)
C10	0.0451 (19)	0.0461 (19)	0.0399 (18)	0.0039 (15)	0.0038 (15)	0.0001 (15)
C11	0.0456 (19)	0.049 (2)	0.0382 (19)	0.0093 (16)	0.0081 (15)	0.0059 (16)
C12	0.053 (2)	0.055 (2)	0.053 (2)	0.0084 (18)	0.0105 (18)	0.0120 (18)
C13	0.070 (3)	0.072 (3)	0.076 (3)	0.010 (2)	0.024 (2)	0.032 (2)
C14	0.093 (3)	0.108 (4)	0.046 (3)	0.025 (3)	0.023 (2)	0.027 (3)
C15	0.088 (3)	0.094 (3)	0.042 (2)	0.026 (3)	0.006 (2)	0.001 (2)
C16	0.057 (2)	0.061 (2)	0.0390 (19)	0.0138 (18)	0.0035 (16)	0.0029 (18)
C17	0.082 (3)	0.062 (3)	0.064 (3)	0.008 (2)	−0.002 (2)	−0.014 (2)
C18	0.069 (3)	0.046 (2)	0.123 (3)	−0.009 (2)	−0.006 (2)	0.024 (2)
O1	0.0467 (13)	0.0490 (13)	0.0387 (12)	−0.0020 (11)	0.0051 (10)	−0.0055 (10)
O2	0.0433 (12)	0.0535 (14)	0.0371 (12)	0.0007 (10)	0.0055 (10)	0.0064 (10)
O3	0.0704 (16)	0.0468 (14)	0.0756 (18)	−0.0126 (12)	0.0120 (13)	0.0075 (13)
O4	0.0708 (16)	0.0526 (15)	0.0419 (13)	−0.0078 (12)	−0.0044 (11)	0.0059 (10)
O5	0.122 (2)	0.0432 (16)	0.086 (2)	0.0179 (15)	−0.0016 (16)	−0.0016 (13)
O6	0.138 (3)	0.083 (2)	0.089 (2)	0.0013 (18)	−0.012 (2)	−0.0349 (17)

### Geometric parameters (Å, °)

**Table:**

C1—O4	1.430 (3)	C10—O2	1.447 (3)
C1—H1A	0.9600	C10—H10A	0.9700
C1—H1B	0.9600	C10—H10B	0.9700
C1—H1C	0.9600	C11—O2	1.373 (3)
C2—O4	1.368 (4)	C11—C16	1.385 (4)
C2—C3	1.376 (4)	C11—C12	1.402 (4)
C2—C7	1.413 (4)	C12—O3	1.366 (4)
C3—C4	1.378 (4)	C12—C13	1.385 (4)
C3—H3	0.9300	C13—C14	1.384 (5)
C4—C5	1.369 (4)	C13—H13	0.9300
C4—H4	0.9300	C14—C15	1.343 (5)
C5—C6	1.396 (4)	C14—H14	0.9300
C5—H5	0.9300	C15—C16	1.412 (4)
C6—C7	1.383 (4)	C15—H15	0.9300
C6—C8	1.479 (4)	C16—C17	1.464 (5)
C7—O1	1.378 (3)	C17—O6	1.209 (4)
C8—O5	1.210 (4)	C17—H17	0.9300
C8—H8	0.9300	C18—O3	1.431 (3)
C9—O1	1.449 (3)	C18—H18A	0.9600
C9—C10	1.489 (4)	C18—H18B	0.9600
C9—H9A	0.9700	C18—H18C	0.9600
C9—H9B	0.9700		
			
O4—C1—H1A	109.5	C9—C10—H10A	110.0
O4—C1—H1B	109.5	O2—C10—H10B	110.0
H1A—C1—H1B	109.5	C9—C10—H10B	110.0
O4—C1—H1C	109.5	H10A—C10—H10B	108.4
H1A—C1—H1C	109.5	O2—C11—C16	119.1 (3)
H1B—C1—H1C	109.5	O2—C11—C12	120.4 (3)
O4—C2—C3	125.0 (3)	C16—C11—C12	120.4 (3)
O4—C2—C7	115.8 (3)	O3—C12—C13	125.5 (3)
C3—C2—C7	119.1 (3)	O3—C12—C11	115.5 (3)
C2—C3—C4	120.7 (3)	C13—C12—C11	119.0 (3)
C2—C3—H3	119.7	C14—C13—C12	120.4 (4)
C4—C3—H3	119.7	C14—C13—H13	119.8
C5—C4—C3	120.7 (3)	C12—C13—H13	119.8
C5—C4—H4	119.6	C15—C14—C13	120.7 (4)
C3—C4—H4	119.6	C15—C14—H14	119.6
C4—C5—C6	119.8 (3)	C13—C14—H14	119.6
C4—C5—H5	120.1	C14—C15—C16	120.9 (4)
C6—C5—H5	120.1	C14—C15—H15	119.6
C7—C6—C5	120.0 (3)	C16—C15—H15	119.6
C7—C6—C8	121.8 (3)	C11—C16—C15	118.6 (3)
C5—C6—C8	118.2 (3)	C11—C16—C17	121.3 (3)
O1—C7—C6	120.2 (3)	C15—C16—C17	120.1 (3)
O1—C7—C2	119.9 (3)	O6—C17—C16	124.3 (4)
C6—C7—C2	119.7 (3)	O6—C17—H17	117.8
O5—C8—C6	123.2 (3)	C16—C17—H17	117.8
O5—C8—H8	118.4	O3—C18—H18A	109.5
C6—C8—H8	118.4	O3—C18—H18B	109.5
O1—C9—C10	113.4 (2)	H18A—C18—H18B	109.5
O1—C9—H9A	108.9	O3—C18—H18C	109.5
C10—C9—H9A	108.9	H18A—C18—H18C	109.5
O1—C9—H9B	108.9	H18B—C18—H18C	109.5
C10—C9—H9B	108.9	C7—O1—C9	114.8 (2)
H9A—C9—H9B	107.7	C11—O2—C10	114.8 (2)
O2—C10—C9	108.3 (2)	C12—O3—C18	117.6 (3)
O2—C10—H10A	110.0	C2—O4—C1	117.4 (2)
